# 3-Dimensional printing and bioprinting in neurological sciences: applications in surgery, imaging, tissue engineering, and pharmacology and therapeutics

**DOI:** 10.1007/s10856-025-06877-4

**Published:** 2025-04-09

**Authors:** Sreejita Dhar, Faraz Ahmad, Aditi Deshpande, Sandeep Singh Rana, Toufeeq Ahmed A, Swagatika Priyadarsini

**Affiliations:** 1 Department of Biotechnology, School of Bio Sciences and Technology (SBST), Vellore Institute of Technology (VIT), Vellore, 632014 India; 2 Department of Bio Sciences, School of Bio Sciences and Technology (SBST), Vellore Institute of Technology (VIT), Vellore, 632014 India; 3 ICAR- National Research Centre on Camel (NRCC), Bikaner, Rajasthan 334001 India

## Abstract

The rapid evolution of three-dimensional printing (3DP) has significantly impacted the medical field. In neurology for instance, 3DP has been pivotal in personalized surgical planning and education. Additionally, it has facilitated the creation of implants, microfluidic devices, and optogenetic probes, offering substantial implications for medical and research applications. Additionally, 3D printed nasal casts are showing great promise for targeted brain drug delivery. 3DP has also aided in creating 3D “phantoms” aligning with advancements in neuroimaging, and in the design of intricate objects for investigating the neurobiology of sensory perception. Furthermore, the emergence of 3D bioprinting (3DBP), a fusion of 3D printing and cell biology, has created new avenues in neural tissue engineering. Effective and ethical creation of tissue-like biomimetic constructs has enabled mechanistic, regenerative, and therapeutic evaluations. While individual reviews have explored the applications of 3DP or 3DBP, a comprehensive review encompassing the success stories across multiple facets of both technologies in neurosurgery, neuroimaging, and neuro-regeneration has been lacking. This review aims to consolidate recent achievements of both 3DP and 3DBP across various neurological science domains to encourage interdisciplinary research among neurologists, neurobiologists, and engineers, in order to promote further exploration of 3DP and 3DBP methodologies to novel areas of neurological science research and practice.

**Graphical abstract:**

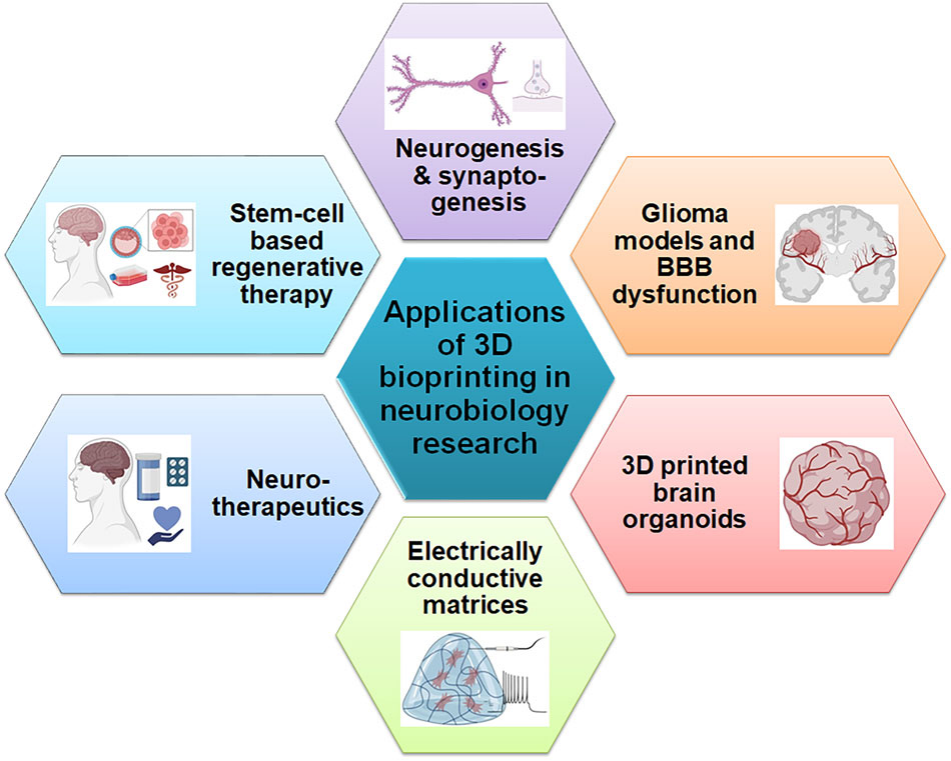

## Introduction

Three-dimensional printing (3DP) serves as a transformative technique that converts digital images into tangible, functional objects [1]. Broadly, 3D printers operate through either additive or subtractive methods—constructing objects by adding material layer-by-layer or by selectively removing layers, respectively. Notably, recent years have witnessed substantial advancements in 3DP, driving its integration into diverse aspects of daily life. The concurrent reductions in the physical footprint, error rates, and costs of 3D printers, coupled with enhanced accessibility to 3D imaging platforms, have facilitated the widespread adoption of this technology, particularly in medical research. Moreover, the exploration and development of novel printing materials (inks) have played a pivotal role in translating 3DP technology into clinical applications [2].

The application of 3DP technology in neurological science research has, in particular, been multimodal and immensely fruitful [3–5]. Complex 3D mimics can be fabricated to accurately represent multiple pathophysiological aspects of disease- and patient-specific human nervous systems. Thus, phantoms for head/skull, cerebrovasculature, spinal cord and nervous tissue have been extensively designed for neurosurgical simulation, planning, training and education [6]. Computer-aided design (CAD) has aided the production of 3D case-specific anthropomorphic phantoms according to the template obtained from clinical imaging, such as computed tomography (CT) and magnetic resonance imaging (MRI) [7]. 3D-printed implants for neuro-regenerative purposes have also been designed and fabricated to aid in the recovery of clinical cases of spinal cord injuries and insults [8]. 3D printed micro-devices and constructs have found immense utilities in animal research for understanding neuronal functions and behavior [9, 10]. In addition, various 3D-printed devices have been shown to reinforce neurosurgical operations in a patient-specific manner.

3DBP and artificial neural tissue engineering are other key facets that have advanced considerably during the last few years [11, 12]. Appropriate biocompatible inks have allowed biomimetic 3D printed acellular scaffolds and substrates upon or into which pertinent cell types can be incorporated in the presence of appropriate chemical signals and factors. Using the three primary technologies; viz., inkjet-based, extrusion-based, and light-assisted printing, biomaterials have been effectively used to produce acellular scaffolds, cell-encapsulated tissues, models of disease, as well as biological platforms for administering or screening drugs [13, 14]. 3DP-mediated fabrication of cell encapsulated ex vivo tissues have considerably enhanced our understanding of complex pathophysiological processes of neurogenesis, synaptogenesis, and gliosis. Fabrication of implantable artificially generated 3D printed neural tissues for stem cell-based therapeutic strategies in spinal cord injuries has also become a viable possibility [15]. Further, 3DBP has shown applications in constructing glioblastoma models, aiding in replicating the tumor microenvironment for mechanistic and therapeutic studies [16]. Organoid research is another area of neurobiology which has hugely benefited from the advances in 3DP, making it easier to follow the pathophysiological mechanisms of the development and progression of neurological diseases, including neurodegeneration and neuropsychiatric conditions [17].

This review strives to offer a current overview of the diverse applications of 3DP and 3DBP in neurological science research and practice. As opposed to previously published reviews which have either assessed the clinical applications of 3DP [3, 6, 8]; or evaluated the biomedical usage of 3DBP technologies [14, 15, 17], in this updated review, we have critically analyzed both these aspects across different neurological science domains. Accordingly, we begin with a brief outline of the widely used 3DP technologies, followed by assessment of their clinical applications in neurosurgery and neuroimaging. Overview of 3DBP and summarization of its biomedical utilities in creating organoids, electrically conductive systems and other fabrications for mechanistic and therapeutic understanding of brain pathophysiological states, such as neuronal injury and gliomas are also undertaken. While not exhaustive, the authors anticipate that this summary will act as a catalyst, drawing multidisciplinary researchers towards developing innovative 3DP- and 3DBP-based research methodologies to advance the understanding, replication, and improvement of therapeutic interventions for neurological disorders.

## Overview of 3DP techniques

**Fig. 1:**
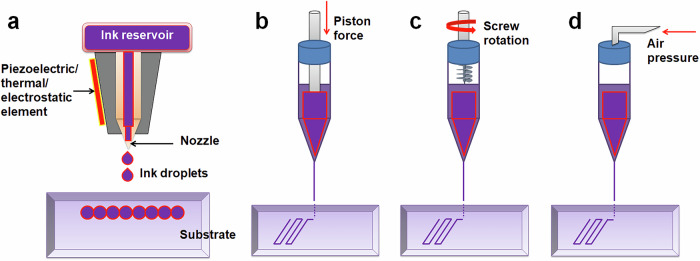
Basic setup for inkjet-based and extrusion-based 3DP. Inkjet-based printing may involve electrostatic, piezoelectric or thermal element **a**. On the other hand, extrusion-based printing involves a piston **b** screw **c** or air pressure **d**

There are several techniques of 3DP which are in widespread use (Table 1). Inkjet-based 3DP (Fig. 1a) is preferred for dispensing inks from a nozzle in a volume-controlled droplet form to produce the desired pattern. Droplets can be applied to recipient substrates using electrostatic, piezoelectric, or thermal methods. In thermal inkjet bioprinting, air bubbles created by focused heating discharge droplets from the nozzle. On the other hand, electrostatic- and piezoelectric-based protocols use pressure generated by the piezoelectric actuator and deflection of the pressure plate in order to eject droplets in a pre-determined pattern. Inkjet bioprinting has several advantages, such as ease of use, low cost, high printing speeds, and fine resolution. However, optimizing relevant parameters to minimize negative impacts on cell viability during the printing process is important. In particular, cell density must be calibrated to avoid excessive shear stresses on cells as they are dispensed through the bioprinter nozzle. A key challenge is achieving an appropriate balance between the desired resolution, material rheological properties, nozzle size, and dispensing speed. While smaller nozzles can enhance resolution, using a size inappropriate for a given bioink’s viscosity increases the risk of nozzle clogging [18].

**Table 1:** Comparison of the commonly used 3DP techniques

3DP technique	Sub-class	Advantages	Limitations	Reference(s)
**Inkjet-based**	Thermal inkjet	High speed, low cost, and precise droplet control	High shear stress (cellular damage), nozzle clogging issues	[18, 19]
	Piezoelectric inkjet	No thermal damage to bioink, fine control over droplet ejection	Potential cytotoxicity from actuator vibrations	
	Electrostatic inkjet	Efficient deposition of low-viscosity bioinks, high resolution	Requires careful control of voltage and charge	
**Extrusion-based**	Pneumatic-driven	Capable of high-viscosity bioinks, simple setup	Pressure variations may impact precision, shear stress may affects cell viability	[19, 20]
	Piston-driven	High control over material flow, capable of printing highly viscous materials	Limited compatibility with shear-sensitive cells	
	Screw-driven	High precise for very high viscosity bioinks	Complex mechanism and expensive	
**Light-assisted**	Laser-assisted	High resolution, minimal impact on cell viability, no nozzle clogging	Expensive and complex setup, limited scalability	[21, 22]
	Two-photon polymerization	Sub-micron resolution, precise structuring for fine features, no nozzle clogging	Slow and limited scalability	
	Digital light processing	Rapid fabrication, good resolution, no clogging issues	Requires photosensitive bioinks	
	Stereolithography	Precise control over layer curing, high structural accuracy, no clogging issues	Limited to photopolymerizable bioinks

Extrusion-based 3DP (Fig. 1b–d) is used for continuous deposition of ink strands through a nozzle, which enables creation of constructs with stronger overall interface integrity than inkjet printing. The two main extrusion-based bioprinting dispensing techniques are based upon pneumatic and physical mechanisms; the latter comprises piston-driven and screw-driven methods. For layer-by-layer 3D structure construction, the stage or ink-filled distribution nozzle is motorized. Pneumatic dispensing is significantly impacted by pressure changes, while piston-driven dispensing typically provides superior spatial control due to the direct influence on the material flow. Screw-driven dispensing is particularly well-suited for extremely viscous materials. Extrusion-based bioprinting exhibits remarkable adaptability, allowing for the utilization of inks with diverse viscosities [19]. Additionally, packaging and bioprinting of cells at reasonably high amounts or in the form of spheroids is possible with this printing approach. In fact, extrusion-based bioprinting remains the most widely used 3DP method for applications in tissue engineering because it can create samples with dimensions, mechanical properties, and cell densities that are physiologically equivalent.

**Fig. 2:**
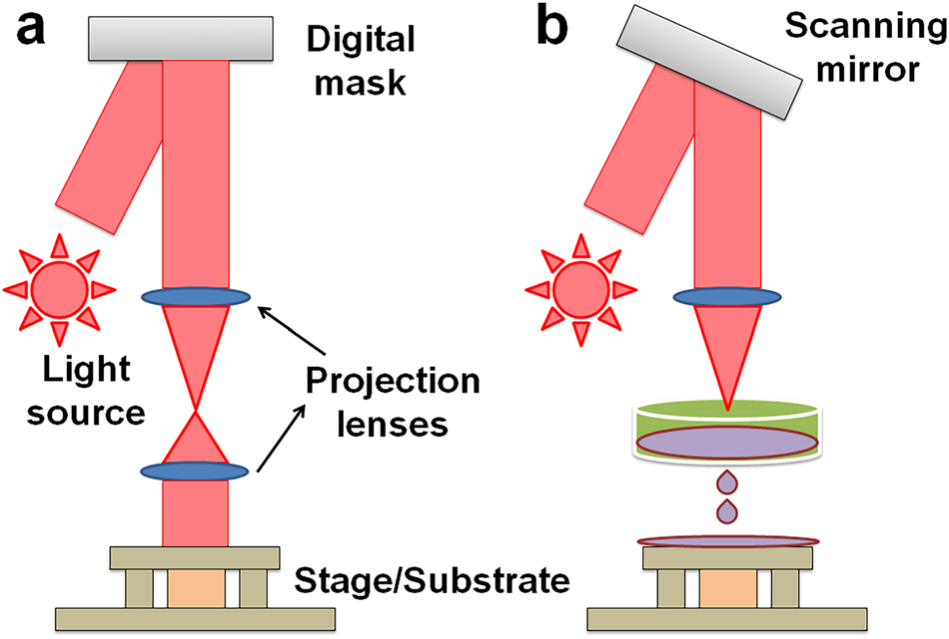
Projection-based **a** and scanning-based **b** light-assisted 3D printing. A laser or other focused light source is used to solidify the photosensitive printing ink. In projection-based printing, a digital light processing (DLP) projector is used to project a 2D image of each layer onto a build stage for selective solidification of the ink as determined by the digital mask. Scanning-based 3D printing uses a computer-controlled scanning system to precisely direct the light to specific locations, allowing for the controlled solidification of the ink in a layer-by-layer fashion

**Fig. 3:**
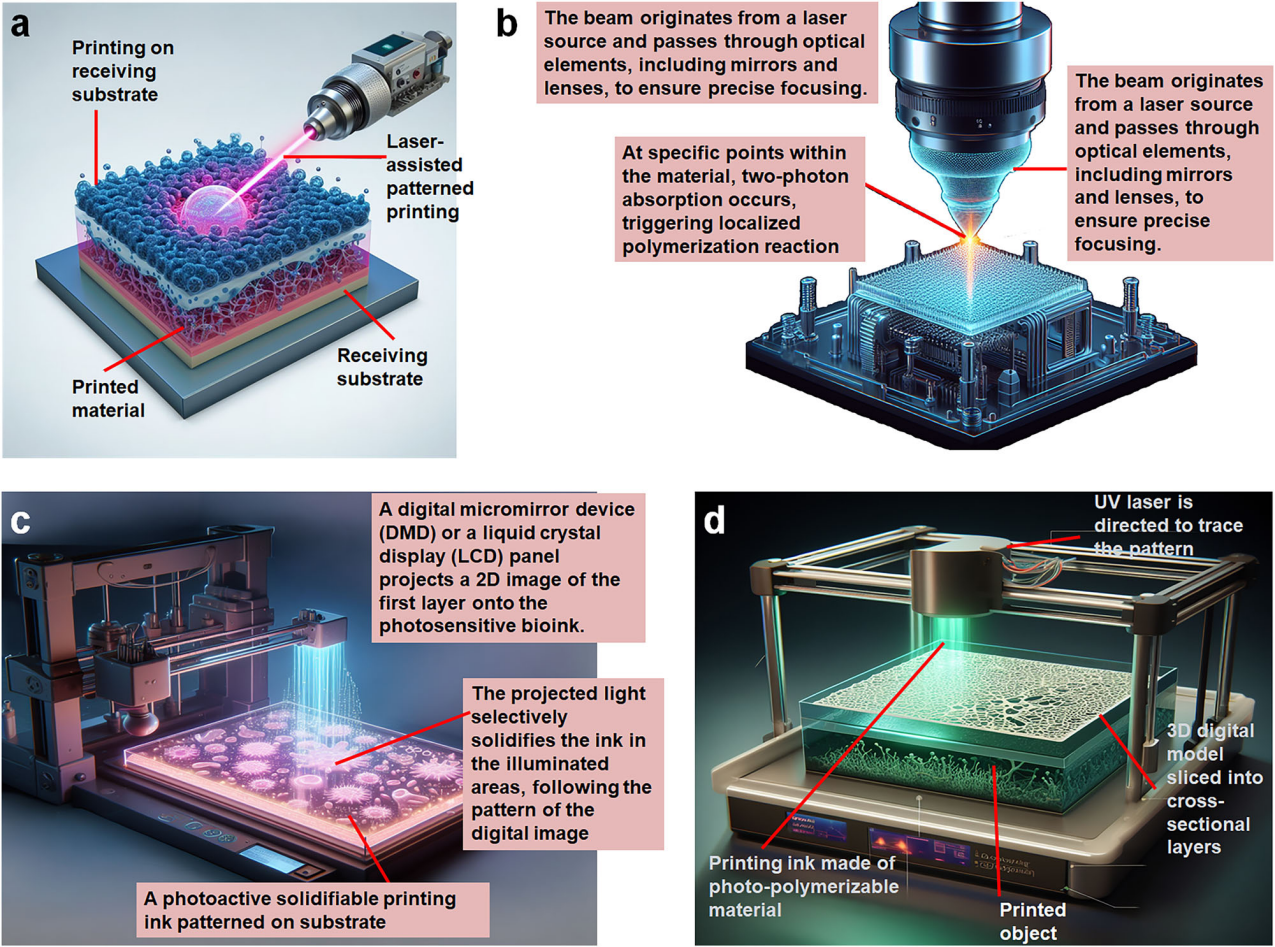
Different versions of light-assisted 3DP. **a Laser-assisted**, the beam originates from a laser source and passes through optical elements, including mirrors and lenses, to ensure precise focusing, allowing patterned printing onto a receiving substrate. **b Two-photon polymerization**, at specific points within the material, two-photon absorption occurs, triggering a localized polymerization reaction. **c digital light processing (DLP)**, a digital micromirror device (DMD) or a liquid crystal display (LCD) panel projects a 2D image of the first layer onto the photosensitive ink, following which the projected light selectively solidifies the ink in the illuminated areas as dictated by the pattern of the digital image. **d Stereolithography**, UV light beam is focused on the printing ink composed of a photopolymerizable material for replication of the desired 3D pattern

**Fig. 4:**
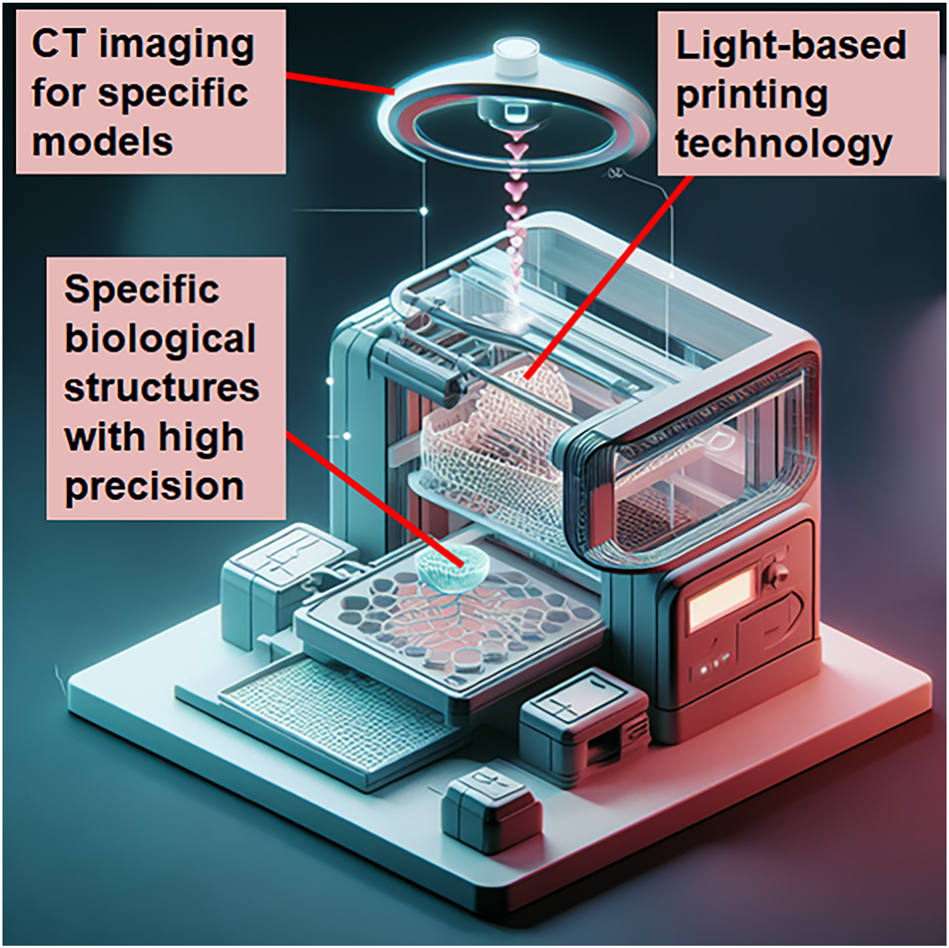
Light-assisted computed axial tomography 3DP. Computed tomography (CT) scanned images are followed to draw desired 3D pattern and précised printing design can be achieved with the help of light assisted technology

Light-assisted 3DP enables photo-polymerization of inks to produce 3D structures. Light-assisted approaches have the advantages of high resolution and accurate oversight of the designs across all three dimensions [20]. Light-assisted 3DP techniques can be categorized into two groups based on the manufacturing processes: projection-based (Fig. 2a) and scanning-based (Fig. 2b). For scanning-based approaches, serial movement along all three axes is often necessary. In order to construct 2D features onto one layer, a donor film or bioinks are first scanned by a laser beam. The third axis, usually the z-axis, is then traversed by the laser beam to produce a 3D structure. In projection-based 3DP, an entire layer is polymerized all at once. Printing usually requires movement along the third axis since one patterned light projection can produce many features on a single plane. Therefore, projection-based strategies often offer higher throughput and quicker printing speeds than scanning-based tactics. With regards to bioprinting, light-assisted 3DP techniques can achieve higher cell viability even for delicate cell types like stem cells in the absence of the high sheer pressures created in inkjet or extrusion bioprinting. For biological applications, two categories of light-assisted approaches are widely used: (i) scanner-based methods, like laser-assisted bioprinting (LAB; Fig. 3a) and two-photon polymerization (TPP; Fig. 3b); and (ii) projection-based methods, such as digital light processing (DLP; Fig. 3c) and stereolithography (Fig. 3d). A laser-assisted 3D printer consists of a pulsing laser source, a receiving substrate, a ribbon with a bioink layer as well as a metal laser-absorbing layer, frequently made of gold or titanium. The laser beam generates droplets of ink to discharge onto the recipient substrate parallel to the ribbon by generating vapor bubbles on the donor substrate layer during the printing process. TPP is a laser-based direct-writing method that uses a rapid laser beam to trace and polymerize the cross-sectional features of 3D structures. TPP polymerizes ink materials by simultaneously taking in two photons generated by a near-infrared femtosecond pulsed laser. Since the diffraction limit of the light source does not restrict TPP’s resolution, submicron size features can be acquired. TPP is suitable for single cell research and fine biomaterial patterning due to the relatively high resolution, but at the cost of a slower bioprinting pace and a constrained ability to scale. Constructions on centimeter- and millimeter-scale can be made using rapid projection-based DLP and SLA 3DP techniques in seconds to minutes. DLP printers frequently come along with a digital micro-mirror device (DMD) chip, a motorized stage or probe, a system of optical paths, and a computer in order to synchronize the movement of the stage or the probe for appropriate pattern generation. Millions of micro-mirrors embedded on the DMD chip can be individually activated or deactivated, allowing for the presentation of intricate patterns with micrometer-scale features. The use of photocurable bioinks ensures polymerization exclusively in regions illuminated by the DMD chip, facilitating the creation of finely detailed architectures with high resolution. This bioprinting technology has been employed to construct functional tissue structures utilizing diverse cell types and extracellular matrix (ECM) elements. DLP bioprinting allows for precise control over the modulus of elasticity and the quantity of biochemical signals, both crucial factors in biological studies. While DLP 3D printing with biomaterials is well-established, certain materials require modification to attain the necessary photosensitivity. DLP technology finds diverse applications, including controlled growth factor release, nerve regeneration, high-throughput drug testing, and the modeling of tissues and diseases [21]. A variant of DLP is computed axial lithography (CAL; Fig. 4), which is a volumetric 3DP technique. This method provides more geometric flexibility, enabling complex non-symmetric 3D structures to be printed. The use of materials with high viscosities eliminates the necessity for supporting materials. The capacity to print around an existing object and the scalability that enables the quick creation of structures on centimeter scale are only a few of this technology’s notable advantages [22].

## Applications of 3DP in neurological sciences

### Neurosurgery

**Fig. 5:**
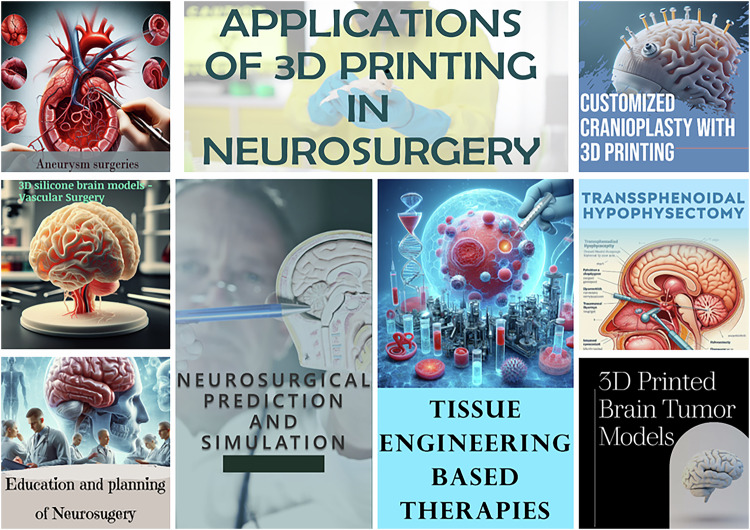
Applications of 3DP in neurosurgery. Utilities include patient-specific model creation for education, predication and planning of different types of neurosurgical procedures. 3D printed micro-devices and implants have also improved neurosurgical outcomes

The tremendous neurosurgical applications of 3DP in education, preoperative planning, and postoperative care have been known for some time now (Fig. 5; Table 2) [23]. Case- and patient-specific 3D models have been routinely created for neurosurgical prediction, simulation, education and planning to benefit clinicians, trainees and patients (reviewed by Blohm and co-workers) [24]. Physical 3D constructs can be effectively fabricated using printing techniques from anatomical information acquired by imaging data for surgical simulations [25], considerably reducing the risk of the subjects undergoing surgeries. This approach has benefited multiple neurosurgical dimensions, including vascular, spine, aneurysm, cranium and tumor/glioma surgeries [26, 27]. The major advantages of constructing 3D printed neurosurgical models lie in their anatomical and textural precision, speed and cost of preparation, and reproducibility and reusability [28]. Not surprisingly, 3D printing has uniquely reproduced patient-specific neuropathologies, particularly those requiring vascular surgeries [29]. Below, we discuss the applications of 3D printing in fabricating patient-specific neurosurgical models and their molds, as well as devices that can potentially aid various aspects of neurosurgical procedures.

**Table 2:** Applications of 3DP in neurosurgery

S. No.	Type of construct	3D printing technique	Applications	Reference
1.	MRI-based patient-specific 3D printed gelatin brain models	Extrusion printing	Realistic appearance and anatomy, resulting in improved patient illustration, neurosurgical training and preoperative planning	[31]
2.	CT image-based MRI-compatible 3D printed ABS segmented head phantom	Stereolithography	Evaluation and refinement of focused ultrasound neurosurgical protocols	[32]
3.	Patient-specific 3D printed ABS aneurysm models based on CT angiography images	Polyjet printing	Training and clipping practice for neurosurgical trainees	[33]
4.	Personalized 3D printed neurosurgical model	Stereolithography	Effective in training for ETV surgery endoscopic third ventriculostomy in hydrocephalic conditions	[34]
5.	Case-specific MRI-based 3D printed silicone vascularized brain models	Stereolithography	Training for neuroendoscopic perception and navigation for ETV surgeries	[35]
6.	Patient-specific 3D printed models of skull base, glioma tissue and fiber tracts, based on data from MRI, CT and DTI tractography images	Multijet printing	Pre-operative preparation for neurosurgical removal of low grade glioma in the frontal lobe	[36]
7.	MRI- and DTI tractography-based case-specific 3D printed model of DLGG in association with subcortical tissue and	Stereolithography	Patient illustration and preoperative preparation for neuro-oncological surgical resection	[37, 38]
8.	Case-specific 3D printed brain tumor models based upon MRI data	Stereolithography	Pre-operative planning for craniotomy procedures	[39]
9.	Patient-specific 3D printed vascular, skeletal and brain lesion models using radiological data	Stereolithography	Preoperative planning and prediction, and patient education for craniopharyngioma, trigeminal neuralgia, and petroclival meningioma cases	[40]
10.	CT and MRI-based patient-specific 3D printed anatomical models of paediatric brain tumors	Material jetting printing	Optimized pre-operative planning for complicated pediatric neurosurgery in neuroblastoma subjects	[42]
11.	3D printed model of hollow cerebrovascular model for a subject with paraclinoid aneurysm	Stereolithography	Preoperative planning and simulation for cerebral aneurysm coil embolization	[43]
12.	Patient-specific PDMS 3D printed cerebrovascular phantom based on CT angiography images	Stereolithography	Cost- and time-effective model of cerebral arterial network for ischemic stroke education and prediction	[44]
13.	Patient-specific 3D printed brain arteriovenous malformation models based upon CT angiography and 3D rotational angiography images	Stereolithography	Accurate representation of arteriovenous malformation, and effective for patient illustration and clinical educational	[45, 46]
14.	3D printed paediatric cerebrovascular disease models using CT angiography data	Stereolithography	Construction of an open-source set of 3D printed models for training and educational purpose of neurosurgeons	[47]
15.	3D printed skull and sagittal sinus model as components of a craniotomy simulator system		Simulating neurosurgical procedures such as bilateral parasagittal craniotomy, dural sinus tear repair, surgery for superior sagittal sinus injury, and correction of air embolism	[49]
16.	CT images-based 3D printed guides with a burr hole in pituitary fossa	Stereolithography	Optimization of transsphenoidal hypophysectomy surgical procedures in dogs	[50]
17.	3D printed personalized PMMA cranioplasty implants	Stereolithography	Cost and time-effective creation of implants improved patient and surgeon satisfaction	[52]
18.	Patient-specific 3D printed PLA skull defect models, and PMMA cranioplasty implants	Stereolithography	Cheap and easy fabrication may for personalized clinical use	[53]
19.	3D printed porous PLA/PLC constructs with PMMA bone cement PCL and PLA-based 3D printed cranioplasty implants	Injection-extrusion printing	Possible utilities in cranioplasty surgeries, particularly for large deformities	[54]
20.	MSCT images-based 3D printed PMMA molds for cranioplasty implants	PolyJet additive printing	Potential applications in personalized cranial reconstruction in TBI cases with large cranial bone defects	[55]
21.	Customized 3D printed silicone reconstruction of skull layer for fabrication of PMMA cranioplasty implants	Material jetting printing	Reliable, secure, easily reproducible and affordable procedure for repairing various skull abnormalities	[56]

*ABS* acrylonitrile-butadiene-styrene, *CT* computed tomography, *DLGG* diffuse low-grade glioma, *DTI* diffusion tensor imaging, *ETV* endoscopic third ventriculostomy, *MSCT* multisliced computerized tomography, *MRI* magnetic resonance imaging, *PDMS* polydimethylsiloxane, *PLA* polylactic acid, *PLC* poly(ε-caprolactone), *PMMA* poly(methyl methacrylate)

Magnetic resonance imaging (MRI) and computed tomography (CT) are commonly utilized to generate imaging data needed to translate patient anatomy into 3D printed models to assist with neurosurgical planning and simulation. However, accomplishing this translation from images to physical 3D printed models presents certain technical challenges [30], which have addressed with significant successes in recent years. For instance, Ploch and co-workers reported 3D printed gelatin constructs as deformable personalized brain models with realistic size, physical appearance and anatomy for improving surgical accuracy and precision [31]. Further, CT scanning data has been translated into a 3D model using acrylonitrile-butadiene-styrene (ABS) ink for the skull and agar-evaporated milk-silica ink for mimicking cerebral tissues. This 3D printed phantom was compatible with MRI and resembled the acoustic characteristics of a real adult brain for evaluation and refinement of focused ultrasound surgical protocols [32]. Wang et al. fabricated acrylonitrile-butadiene-styrene 3D printed aneurysm models using CT angiography data of several patients with middle cerebral artery aneurysm for training and clipping practice of neurosurgical trainees [33]. Interestingly, complementation of Hollywood-style specific effects with 3D printing has been elicited in the fabrication of realistic neurosurgical models which can be suitably and efficiently used for training of endoscopic third ventriculostomy in hydrocephalic conditions [34]. More recently, Ramirez and colleagues reported the construction of case-specific 3D printed molds using patients’ MRI data, which were used to accurately fabricate 3D printed silicone brain models in which blood circulation was recreated using paprika oleoresin dye-containing fluid. These constructs were found to replicate realistic situations for training the surgical procedure of endoscopic third ventriculostomy [35]. Patient-specific 3D printed models of the skull base, glioma tissue, and fiber tracts have been successfully used for preparative purposes for complex neurosurgical operation in a clinical case with low grade glioma in the frontal lobe [36]. Similarly, 3DP has been employed to fabricate models of diffuse low-grade gliomas from data derived from functional neuroimaging studies in clinical cases. Such models accurately recapitulated the 3D structure of the glioma tissue with their spatial and anatomical relationships to white matter tract and cortical and subcortical pathways, indicating their potential utilities in preoperative planning and physician-patient communication for neuro-oncological surgical resection procedures [37, 38]. More recently, Dho et al. have reported the establishment of a cloud-based interactive platform for quick fabrications of patient-specific 3D printed brain tumor models and found these models to be significantly better than conventional MRI data in neurosurgical planning for surgical procedures and craniotomy design [39]. 3D printed models accurately representing the anatomical and physical characteristics of vascular, skeletal and brain lesion attributes in a case-specific manner have been also fabricated for preoperative planning and prediction in clinical cases of petroclival meningioma, trigeminal neuralgia and pediatric craniopharyngioma [40]. Of note, because of their complexity and strenuousness, pediatric neurosurgical procedures may immensely benefit from the applications of 3D printing in anthropomorphic modeling of brain and its constituents (reviewed by Karuppiah and cowrokers) [41]. In this regard, complementation of 3D modeling (such as computer-aided design, and virtual and augmented reality) techniques with 3DP may serve as trend-setters in pediatric neurosurgery [42].

A stereolithography-based 3D printed model of cerebrovasculation was reported as an efficient tool for preoperative planning and simulation of the surgical treatment procedure of cerebral aneurysm coil embolization in a 66 year old woman [43]. Likewise, using the images from CT angiography from a subject with symptomatic carotid stenosis, Nilsson et al. created complex phantoms of cerebral arterial trees using polydimethylsiloxane polymer using a stereolithography 3DP. They proposed that this time- and cost-efficient strategy for fabrication of 3D cerebrovascular phantoms may aid in reproducing patient-specific morphological details for studying and predicting ensuing ischemic strokes or transient ischemic episodes [44]. Similarly, 3D printed custom-made pathological models of brain arteriovenous malformations have been proposed as efficient educational tools for replication of the sizes and anatomical attributes of the lesion sites with high fidelity, accuracy and precision. Such models generated from CT angiography and 3D rotational angiography studies have been proposed to aid surgical education among trainees, preoperative planning by neurosurgeons, and subjective understanding in the patients and their relatives [45, 46]. Graffeo et al. have utilized previously acquired imaging data from multiple subjects with pediatric cerebrovascular lesions, including arteriovenous and vein of Galen malformations and aneurysms to construct an open source set of 3D printed models for training and education purposes [47]. Open source and free-to-use software for the 3D printing of neurosurgical models have also been designed and submitted recently by Vezirska et al. [48]. Lastly, 3D printing has applications in modeling unfamiliar and unexpected scenarios, such as the complications in neurosurgical operations. Thus, Cleary and co-workers used 3D printing techniques to fabricate a brain and skull model to imitate the complication of air embolism during surgery for superior sagittal sinus injury. The simulation was used for training and testing the effectiveness of trainees to handle stress during operative procedures. Further, the participating trainees concurred that this strategy was effective and realistic in recreating stress of neurosurgical procedures [49].

Further, advances in 3DP technology have led to invention of devices which aid and ease neurosurgical procedures. For example, 3D printed patient-specific guides for fine-tuning operative procedures of transsphenoidal hypophysectomy have been constructed and validated in dogs [50]. Another widely used application of 3DP is in cranioplasty which is a surgical procedure to restore and repair birth-acquired or physical injury-induced irregularities in the skull. Advances in computer-aided design (CAD) and 3DP techniques have proved to be paradigm shifters in the design of patient-specific implants made of various polymers (e.g., poly-methyl methacrylate) and metallic material (e.g., titanium) (reviewed by Bonda and colleugues) [51]. These 3D implants not only accurately match and integrate with the exterior of the skull but also provide precise mechanical force distribution. In addition, these implants may be designed to facilitate tissue engineering-based therapies by inducing the capacity to seed, grow and allow the differentiation of allogenic bone progenitor stem cells. In the recent literature, one can find several examples of applications of 3DP for cranioplasty prostheses. For example, Morales-Gómez and colleagues employed 3D printed customized poly-methyl methacrylate implants in 22 subjects undergoing cranioplasty, showing their effectiveness, durability and acceptability [52]. In addition to poly-methyl methacrylate, which remains one of the most cost-effective 3D printed materials for cranioplasty prosthesis [53], poly-ε-caprolactone (PCL) and polylactic acid (PLA) have also been evaluated for their potential usage as bone cement for 3D printed scaffolds for restoration of large defects in the cranial bone [54]. Of note, in addition to cranial implants, cranial defects have also been modeled using 3DP in order to get precise information on size and shape requirements preoperatively. Such models can be fabricated using PLA on a 3DP and can then be used to fabricate molds for shaping the implant itself. Strategies for preoperative design of 3D printed cranioplasty implants based upon patient-specific prefabricated 3D printed skull molds have been successfully implemented in traumatic brain injury clinical cases following decompressive craniectomy [55]. Similarly, Csámer et al. have recently used the 3D reconstruction of CT skull scan to superimpose the intact half of the cranial bone onto the defective side for the precise size and shape measurement of the cranioplasty implant. They then fabricated a silicone mold which was used to 3D print the poly-methyl methacrylate implants for multiple cranioplasty subjects, providing support for the strategy’s safety, reproducibility, and reliability [56].

### Micro-devices and micro-fabrications

**Fig. 6:**
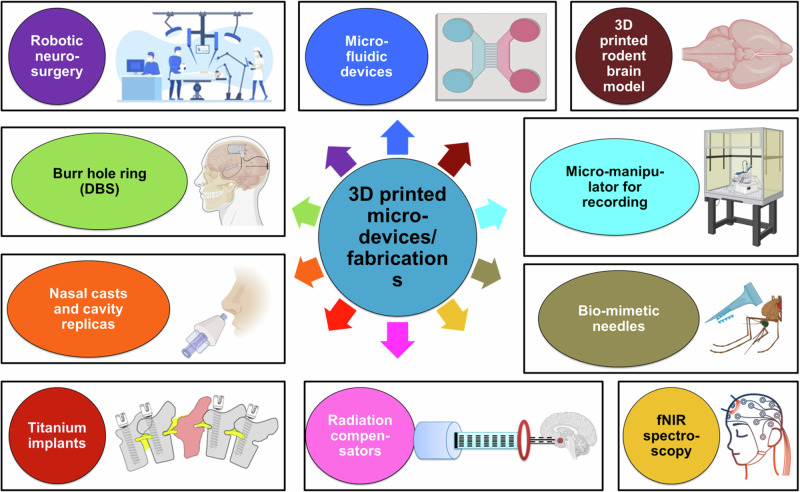
Utilities of 3D printed fabrications in neurological sciences. Applications vary from clinical uses in creation of burr hole rings, nasal casts and implants to construction of microfluidic devices and micromanipulators for preclinical studies

In recent years, there have been several instances of applications of 3DP for fabrication of devices which aid in therapeutic and research aspects of neurological sciences (Fig. 6; Table 3). We begin our discussion with implants. 3D printed titanium-based custom made implants have been proposed as a robust orthopedic tool for spinal reconstruction following tumor resection [57], and intralesional extracapsular excision [58]. Such 3D printed customized implants have also been evidenced to aid spinal reconstruction after successful surgical removal of primary spinal tumors (reviewed by [59]). Indeed, Wei et al. have provided evidence for the integration, reliability and durability of titanium-made 3D implants for spinal reconstruction after resection to remove tumors in the upper cervical segments in a case-specific manner [60].

**Table 3:** 3D printed devices/micro-devices and their applications in neurological sciences

S. No.	Micro-device	3DP technique	Utilities	Reference
1.	Customized patient-specific titanium alloy-based 3D printed vertebral body implants	Electron beam melting	Reliable upper cervical spine restoration with long-term stability after primary tumor removal	[60]
2.	3D printed biomimetic (resembling honeybee stingers) PVC surgical needles	Polyjet printing	Reduced frictional insertion force for insertion in bovine brains, with potential utilities in brain stimulation and biopsy techniques	[61]
3.	3D printed burr hole ring for skull fixation device		Enhanced fixation strength for steady fixation during deep brain stimulation	[62]
4.	3D printed wearable patch for fNIRS system		Component of a cost-efficient 10-channel wireless functional NIR system, replacing the requirement of traditional, and often uncomfortable wired electrode system	[63]
5.	3D printed control unit, detachable optodes and detector holders for fNIRS system, “WearLight”		Components of the “WearLight” fNIRS system based upon Internet-of-Things for real time brain function assessment	[64]
6.	Polyamide nylon-based mesh like 3D model of cerebral cortex	Selective laser sintering	Reproduction of brain surface and some deep structural anatomy for planning and preparation for intracranial EEG electrode implantation	[65]
7.	3D printed plastic compensators for cobalt-60 radiotherapy	Stereolithography	Component of a cost-effective device for planning and delivering radiotherapy for head-and-neck metastases	[66]
8.	3D printed components of MRI-guided “MINIR-II” neurosurgical robot		Important components (linking mechanisms, connectors, gears, pulleys) result in eased operation of the robotic system	[67]
9.	3D printed nasal replicas		Assessment of deposition patterns of drugs delivered via the nose-to-brain route, indicating the suitability of electrically guided delivery systems	[69]
10.	Customized 3D printed nasal replica casts		Investigation of deposition pattern following drug administration in a patient-specific manner	[70]
11.	3D printed rat skull-nasal cavity model	Polyjet printing	Evaluation of the deposition of Evans blue dye as a test delivery chemical	[71]
12.	3D printed human nasal cavity model	Stereolithography	Nasal deposition studies for anti-epileptic, diazepam-containing nanoparticles	[72]
13.	3D printed human nasal cavity replicas	Stereolithography	Assessment of nasal deposition pattern of therapeutic, donepezil	[73]
14.	3D printed model of mouse brain for cyro-sectioning	Polyjet printing	Affordable and precise technique for cryopreservation and subsequent cryosectioning of fixed brain hemispheres	[75]
15.	3D printed recording chamber and micro-manipulators	Stereolithography	Increased efficiency of voltage-sensitive dye-based imaging for brain network activity	[76]
16.	Customized 3D printed slice chamber components		Increased efficiency of perfusion, and precise manipulation of acute mouse brain slices for the investigation of neuronal activity patterns	[77]
17.	3D printed recording micro-device for holding movable micro-drives	Stereolithography	Eased simultaneous measurement of electrical activity in hundreds of individual neurons in live rodents	[78]
18.	3D printed micro-manipulator device for micro-wire electrode implantation	Stereolithography	Investigation of activity in utricular afferents in the inner ear of oyster toadfish in response to auditory stimuli	[79]
19.	Paxinos coordinate system-based 3D printed PLA head mounts for mice, and customized hippocampal guide cannulas	Fused deposition modeling extrusion printing	Administration of oligomeric amyloid beta species to hippocampal CA1 regions	[80]
20.	3D printed PDMS microfluidic devices for targeted delivery of chemicals	Photolithography	Assessment of axon growth cone responses to patterned deposition of trophic molecules such as ephrinA5	[81]
21.	3D printed objects with specific attributes to generate tangible stimuli	Inkjet printing	Study objects for study of visual and somatosensory perception in humans 83,84,85	[83]
22.	3D printed complex naturalistic objects such as “digital embryos”	Conventional 3D prototyper	Perceptual learning of the processes of embryogenesis, morphogenesis and phylogenesis	[84]
23.	3D printed transparent natural scene-based textured surfaces	Material jetting printing	Study of textural perception and discrimination	[85]
24.	3D printed textured surfaces of bowls containing rewards	Extrusion printing	Assessment of textural learning in rodents	[86]

*fNIRS* functional near infra-red spectroscopy, *NIR* near infra-red, *PLA* polylactic acid, *PMDS* polydimethylsioxane, *PVC* polyvinyl chloride

Instances of 3D printed devices/micro-devices to aid therapeutic protocols against brain disorders are also present in the literature. For instance, Sahlabadi and co-workers fabricated 3D printed biomimetic surgical needles using the honey bee stinger template to regulate the insertion force in order to control the damage to soft tissues like brain and liver. Upon testing the 3D printed needles in bovine brain tissues, they reported reduced insertion force by up to 25%, indicating their potential utilities in varied applications such as brain stimulation, biopsy and brachytherapy [61]. 3DP has been employed to construct burr hole ring of the fixative device used for deep brain stimulation in human subjects, with potential applications in therapies against traumatic brain injuries [62]. Furthermore, Abtahi and colleagues have fabricated a 3D printed wearable patch which can relay hemodynamic responses of the brain for functional near infrared spectroscopic (fNIR) studies wirelessly, eliminating the need for complex, uncomfortable wiring associated with the conventional electrode system [63]. They further developed this prototype into “WearLight”, a wearable fNIR spectroscopy system with 3D printed detectors and optodes to encapsulate near infrared light sources [64]. Javan et al. created a nylon-based mesh like 3D model of cerebral cortex recapitulating the surface and deep structure anatomy for pre-procedural preparation of intracranial electroencephalography (EEG) electrode placement. The model was cheap, durable and potentially suitable for identification of seizure foci in epilepsy patients requiring EEG monitoring [65]. Another area where 3DP has made a mark is radiotherapy for neuro-oncological purposes. Thus, Oh and colleagues fabricated 3D printed compensators for delivering high intensity-modulated radiations in the form of cobalt-60 beams, which could benefit low/middle-income economies because of their efficiency, reliability and cost-effectiveness [66]. Interestingly, efficacy of MRI-guided robotic neurosurgical procedures may also benefit immensely from 3DP techniques, as evidenced by the design and evaluation of a multi degree-of-freedom MINIR-II robot [67].

Lastly, 3DP has elicited immense potential in designing custom-made nasal casts, which can be utilized to optimize drug administration to the brain by bypassing the blood brain barrier (BBB), via the olfactory route (reviewed by [68]). However, there are anatomical (e.g., structure of the nasal valve and turbinate) and functional (e.g., identification of appropriate drug dose to alleviate side-effects, non-specificity and increase bioavailability) challenges associated with nose-to-brain delivery of drugs. Further, subtle changes in the geometries and physical dimensions of nasal cavities depend on the subjects’ ages, genders and ethnicities. Recently proposed approaches in customized design of 3D printed nasal casts may circumvent some of these problems. For example, electrical guidance of charged therapeutic particles may limit drug loss and stimulate their deposition over the nasal epithelial. Indeed, 3D printed nasal replicas have been employed to test the efficacy of electrically guided nose-to-brain delivery of charged powdered aerosolized material [69]. Warnken et al. have used the CT scan data of 10 individuals (5 children and 5 adults) to fabricate customized 3D printed nasal replicas to study the deposition patterns of test nasal spray in order to optimize the angle of dosing in a case-specific manner [70]. 3D printed rat skull-nasal cavity models have been employed by Bors et al. to initial test the deposition of Evans blue dye as a test deliverable. Using the information, the authors further reported that PSC-833-mediated inhibition of p-glycoprotein (which extrudes exogenous chemicals from the body) prior to nasal delivery of the test drug, quinidine increased the brain availability of the latter [71]. More recently, nose-to-brain delivery of anti-epileptic therapeutic diazepam in form of nanostructured lipid carrier hydrogel has been evaluated in 3D printed human nasal cavity casts. Optimizations of physical properties of the drug formulation, such as particle size, zeta potential, rheology, polydispersity index, and dosing parameters, as well as optimization of the choice of the dosing device, airflow and angle of administration, were efficiently performed for increasing nasal deposition and reducing drug loss [72]. 3D printed nasal cavity replicas have also been employed to investigate the potential efficacy of nose-to-brain delivery of donepezil, a therapeutic implicated for the management of Alzheimer’s disease [73]. The authors used an optimized formulation of donepezil in form of a chitosan-based sprayable thermogel for increased biocompatibility and release, which may serve as a template for the design of other neurotherapeutic formulations for evaluation in 3D printed models and subsequently for optimized nose-to-brain delivery in vivo. While studies using 3D printing of nasal cavity replicas have stimulated our hopes of exploiting the nasal route of brain drug delivery, confirmation in vivo models and under clinical settings is warranted before this technique can enter widespread usage.

Design of cheap and precise lab-ware 3D for research studies, particularly for neurological sciences, has become convenient for the first time since the advent of accurate, cheap and size-efficient 3DP [74]. For instance, Pinskly and co-workers fabricated a 3D printed model of mouse brain to design cavities in molds used for cryopreservation and subsequent cryosectioning of fixed brain hemispheres. The model increased the efficiency of obtaining precisely aligned slices for histology-based neuroanatomical evaluation of mice brains, which was reusable and could be attuned to age and species [75]. Similarly, a 3D printed recording chamber with a micro-manipulator has been designed recently, allowing for the appropriate positioning of a carbon fiber microelectrode array capable of simultaneously stimulating and recording multiple neurons. In combination with voltage sensitive dye imaging, the recording chamber with its micro-manipulator could prove to be a game changer in direct modulation of electrical activities of multiple neurons simultaneously, while concomitantly recording their activities ex vivo [76]. Similarly, 3DP has been employed to fabricate a custom-made slice chamber for holding, perfusing and precise manipulation of mouse brain slices in order to study neuronal networks using optical imaging [77]. As opposed to these ex vivo applications, Kloosterman et al. devised 3D printed recording micro-device for holding high density array of tetrode micro-drive arrays able to simultaneously measure the electrical activity in hundreds of individual neurons upon implantation in freely moving rats [78]. Similar 3D printed micro-manipulator devices with microwire electrodes have also been employed for studying the responses of utricular afferents in oyster toadfish inner ear in response to environmentally-relevant auditory stimuli, such as con-specific sounds [79]. Tropea and colleagues have recently employed 3DP to fabricate Paxinos coordinate system-based head mounts for mice and customized guide cannulas for accessing hippocampal CA1 regions. The 3D printed devices made of PLA polymers were found to be suitable for assessment of object recognition memory under in vivo settings. Further, the appropriate localization of the guide cannulas was tested using methylene blue and Nissl staining [80], signifying the applications of 3DP in manufacturing customized cannulas for accessing other brain regions for neurobiology research in animal models.

3D printed microfluidic devices have been known for their applications in guiding precise patterns of axonal outgrowth for some time now. This ability is because specific axon guidance trophic molecules can be specifically printed in designated patterns to drive axonal outgrowth [81, 82]. In addition to the applications in molecular and cellular studies, systems neuroscience has also benefited from the advances in 3DP. Systems neuroscience deals with the structure of neural circuitry and systems for multiple functions, including sensory-motor and visual perceptions. In recent years, 3D printed objects with specific attributes have been constructed as complex perceptual stimuli not normally found in nature, which can potentially be used to understand the response elements in the brain involved in perception and perceptual learning of shape [83, 84] and texture [85]. In rodent studies, 3D printing has been applied to construct surfaces with specific textures to study whisker/barrel cortex-dependent textural judgment and learning in freely moving rodents [86].

### Neuro-imaging and anthropomorphic phantoms

Phantoms in radiometric and magnetic resonance imaging refer to reference objects created to mimic human tissue. Such anthropomorphic phantoms can be created using liquid and gel substances and are becoming increasingly popular in neurological studies for refining surgical, imaging and implantation studies (reviewed by [87]; Table 4). 3D slicer tools and printing are increasingly used to transform medical images into 3D models and then to print them in a precise manner and with the appropriate ink material [88]. A plethora of 3DP techniques have been used to fabricate anthropomorphic phantoms for CT and MRI imaging purposes [89]. These include photo-curing 3DP (e.g., stereolithography, polymer jetting and DLP) [90], fused filament fabrication (FFF) or fused deposition modeling (FDM) [91], which involves deposition of melted thermoplastic materials such as ferromagnetic PLA, and inkjet-based 3DP of stacked printed papers composed of radiopaque ink [92].

**Table 4:** Applications of 3DP in creation of phantoms for neuro-imaging

S. No.	Construct	3DP technique	Applications	Reference
1.	3D printed PMMA brain phantoms using CT data	Stereolithography	Potential utilities in obtaining customized reconstructions for studying anatomical changes in pathologies	[90]
2.	3D printed mouse brain phantoms using CT scan data	Fused deposition modeling printing	Applications in thermal ablation and transcranial focused ultrasound studies	[91]
3.	Paper-based anthropomorphic 3D printed CT phantoms	Stereolithography	Emphasizes potential for simulation of patient imaging and assessment of CT dosage and image quality	[92]
4.	3D printed biphasic alginate-agarose-carrageenan-based anthropomorphic brain phantoms	Extrusion printing	Assessment of MRI protocols	[93]
5.	3D printed brain phantoms made up of electrically conductive carbon nanotubes	Stereolithography	Adjustable electrical conductivity of phantoms has applications for optimization of TMS protocols	[94]
6.	3D printed acoustically relevant skull model with a vascular phantom	Stereolithography	Utilities for improving TUI and transcranial focused ultrasound techniques for humans	[96]
7.	3D printed brain and skull phantoms of appropriate anatomical and acoustic characteristics	Combination of fused deposition modeling and liquid crystal display photolithography printing	Applications in TUI studies	[97]
9.	3D printed brain and skull phantom composed of a skull-like and multiple brain tissue-like layers	Extrusion printing	Potential uses for improvements in TUI techniques for humans	[98]
10.	PDMS-based 3D printed photoreceptor phantoms	Two-photon polymerization	Evaluation of adaptive optics imaging systems	[99]
11.	3D in vitro brain AVM model using 3D rotational angiography images	Fused deposition modeling	Recreation of anatomical characteristics of nidus in a case-specific manner allows training and evaluation of embolization techniques and devices	[100]
12.	Customized 3D printed anatomically relevant models of pediatric cerebrovascular lesions using MRI and CT data	Stereolithography	Reduction in neurosurgical times for pediatric cases of AVM and VOGM	[101]
13.	3D printed in vitro carotid artery aneurysm models	Material jetting printing	Mimics carotid artery malfunctions and changed blood flow, which can be employed for high-speed angiography studies	[102, 103]
14.	3D printed brain phantoms		Simultaneous assessments of motion sensitive MR brain imaging techniques	[104]
15.	3D printed anthropomorphic brain phantom mimicking the T1/T2 relaxation characteristics	Stereolithography	Utilities as an MRI reference object for brain imaging	[105]
16.	Real-sized 3D printed brain models based upon MRI data	Stereolithography	Utilities in accurately representation of lesion size and volume loss in pediatric cases of hypoxic ischemic injury	[107]
17.	3D printed head and brain phantoms		Human brain-like dielectrical properties allow brain stroke imaging studies	[109]

*ASA* acrylonitrile styrene acrylate, *AVM* arteriovenous malformation, *CT* computed tomography, *MRI* magnetic resonance imaging, *PDMS* polydimethylsiloxane, *PMMA* polymethyl methacrylate, *TES* transcranial electric stimulation, *TMS* transcranial magnetic stimulation, *TUI* transcranial ultrasound imaging, *VOGM* vein of Galen malformation

**Fig. 7:**
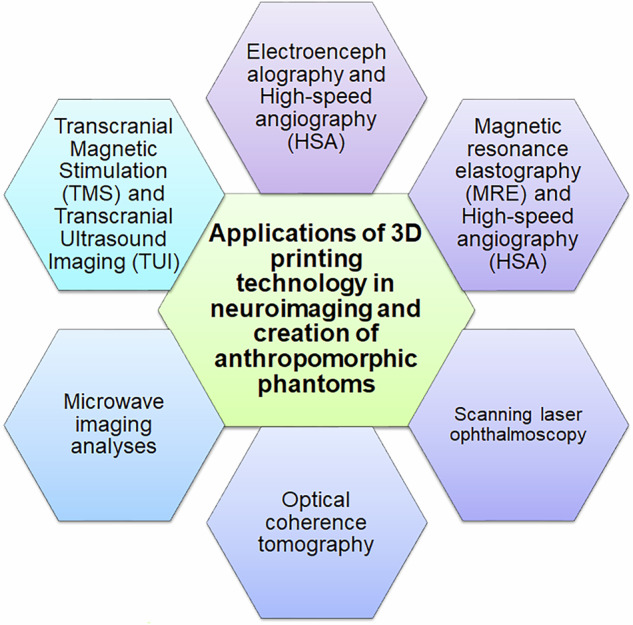
Applications of 3D printing in neuroimaging. Numerous possibilities for aiding microwave imaging, ophthalmoscopy, transcranial magnetic stimulation (TMS) and ultrasound imaging (TUI), magnetic resonance elastography (MRE), and high-speed angiography (HAS) have been advocated

Several examples of 3D printed phantoms resemble the anatomy, texture and relaxation times, and electromagnetic attributes of the real brain for easing the performance of imaging studies in patients (Fig. 7). For example, extrusion 3DP has been used to fabricate alginate-agarose-carrageenan-based anthropomorphic brain phantoms to mirror the differences between white and gray matter to improve MRI scanning of brain tissues [93]. Other recent applications of 3DP in the construction of brain phantoms are related to techniques such as EEG and transcranial magnetic stimulation (TMS), which require the ability of the phantoms to mimic the electrical conductivity of the white and gray matter [94, 95]; and transcranial ultrasound imaging (TUI), which necessitates the phantoms to be able to recapitulate the acoustic features [96]. Recently, Denis and co-workers have constructed a phantom using polyvinyl and PLA using a combination of FDM and LCD photolithography-based 3DP, which resembles real brain tissues for the benefit of refining the protocols involved in TUI [97]. It has also been proposed that full-waveform inversion reconstructions can be exploited for appropriate fabrications of 3D printed phantoms to allow better application of TUI in clinical settings [98].

Optical coherence tomography and scanning laser ophthalmoscopy also fall in the growing list of imaging techniques that may benefit from 3DP technology. Indeed, Kedia et al. have fabricated polydimethylsiloxane-based 3D phantoms resembling outer segments of retinal cone photoreceptor cells with potential utilities in aiding assessment of anatomically relevant lateral resolution by adaptive ophthalmic imaging techniques [99]. Another recent application of 3DP is in modeling cerebrovascular anatomical structures using the imaging data obtained, for instance, from 3D rotational angiography. Such models may allow precise replication of cerebrovascular functional measurement in a patient-specific manner to evaluate any deficits [100]. Interestingly, 3D printed models of cerebrovascular lesions have also been developed to mimic the arteriovenous malformations in pediatric brains, which could aid in CT and MRI studies in a patient-specific manner [101]. Similarly, carotid artery malfunctions and altered blood flow have been modeled using 3D printed in vitro constructs, which can be used for high-speed angiography (HSA) studies in aneurysms [102, 103]. Lastly, a recent report indicates that magnetic resonance elastography (MRE) studies of brain tissues may benefit from 3D printed phantoms that mimic the endogenous low frequency intrinsic motion [104]. Thus, it is clear that while significant challenges remain (as an example, refer to the study by Kraft and co-workers; [105]), 3DP continues to contribute to our advancements in multiple types of imaging techniques used to study the brain and its functional attributes [106].

Lastly, Chacko and colleagues have recently proposed using 3D printed brain models transformed from MRI data for visual and tactile presentation of the pathological findings of imaging studies. For this purpose, they fabricated real-sized 3D brain models which accurately depicted the size of the lesions and tissue volume loss [107]. Further, the investigators argued that as opposed to textual and 2D images, such 3D printed representations may increase perceptual sensitivity and specificity of identification of neuropathological conditions, such as cortical and basal ganglia-thalamic hypoxic-ischemic injuries by radiologists, non-radiologist clinicians and legal professionals [108]. Similarly, 3D phantoms with dielectric properties resembling the human brain have been printed to aid microwave imaging analyses [109].

## 3DBP and neuroscience research

Bioprinting refers to printing living cells encapsulated into printable bioink materials. Bioprinting of cells in a tissue- or organ-like arrangement requires a bioactive scaffold, which acts as a rudimentary extracellular matrix, into or onto which cells can be printed. The idea is to derive morpho-functional support from the scaffold in order to allow cells to reside, grow, differentiate and mature. Such systems have shown relatively high potential for regenerative therapy in orthopedic research [110]. However, their applications in neurological research have been somewhat retarded, possibly due to difficulties in inducing neuronal induction, differentiation and formation of intricate 3D communicative networks.

One of the most important factors to consider in bioprinting is the selection of bioinks [111, 112]. Bioinks must maintain the physiological extracellular matrix-like environment and physical properties, while at the same time be able to support neuronal differentiation, maturation and survival. Further, bioink materials incorporated with cells should be printable with minimal reduction in cellular viability during the process of 3DP. Physico-chemical conditions for 3D bioprintability of bioinks must be standardized and characterized thoroughly. To be used successfully, bioinks have to be deformable as well as flowable and be able to maintain their stability and desirable shape after printing. Assessments of rheological properties (e.g., viscosity and shear thinning) must hence be performed for optimal results [113]. Further, biological characterization should be described in terms of biocompatibility and biodegradability. Additionally, they should elicit capabilities of structural maintenance compatible with living, functional cells and be able to withstand the period of cell culture or in vivo biological conditions, regardless of the crosslinking techniques used [114].

**Fig. 8:**
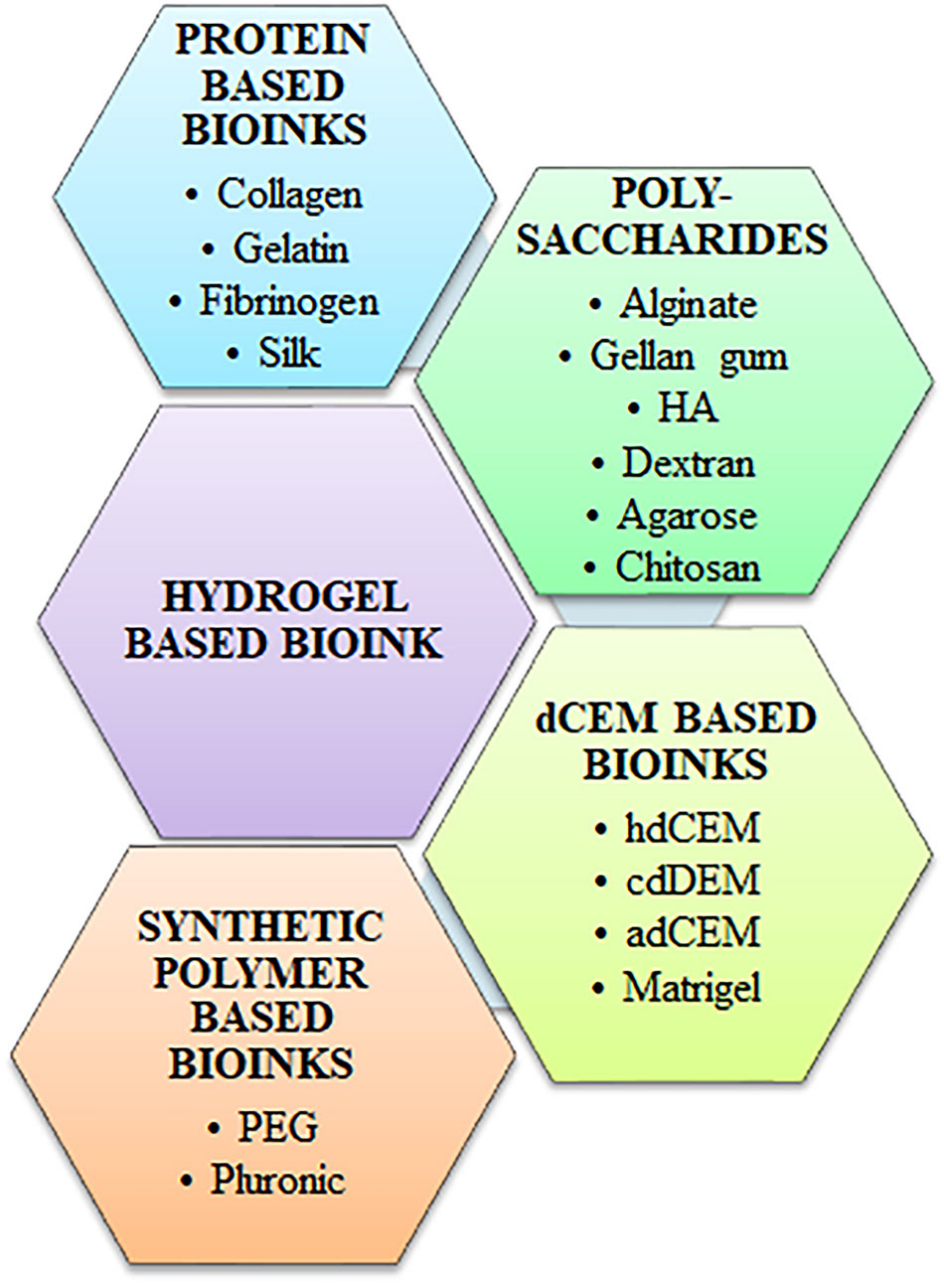
Various bioinks for 3D bioprinted hydrogels used in neurobiology. Bioinks may be based upon proteins, polysaccharides as well synthetic polymers

Recent studies indicate that it is possible to envision 3D printed scaffolds made of different bioinks and appropriate inductive cues that allow precise arrangement of multiple functional cell types, similar to the native brain [115]. Biocompatible hydrophilic polymers elicit tremendous advantages in these aspects. Indeed, biocompatible hydrogels have been shown to have excellent potential for supporting cell soma while allowing the extension of neurites in differentiated neural cells. Such neural tissue engineering approaches have tremendous implications for studying disease mechanisms and evaluating therapeutic agents [116]. While synthetically derived hydrogels can be functionalized with bioactive chemicals, hydrogels based on naturally occurring biopolymers are preferred because the goal is to make a biomimetic microenvironment [117]. There are numerous advantages to using hydrogels from natural materials or their derivative as tissue scaffolds. For instance, they have specialized cell-binding sites that are advantageous for the attachment of cells, and their spreading, development, and differentiation. They are also often biodegradable and biocompatible. Additionally, they can be functionalized using bioactive chemicals such as neurotrophins, growth factors and other signaling molecules, which aid in promoting cellular differentiation and maturation in a cell type-specific manner. Figure 8 shows some of the common materials used as bases for hydrogels in 3DBP of neuronal-like cells.

**Fig. 9:**
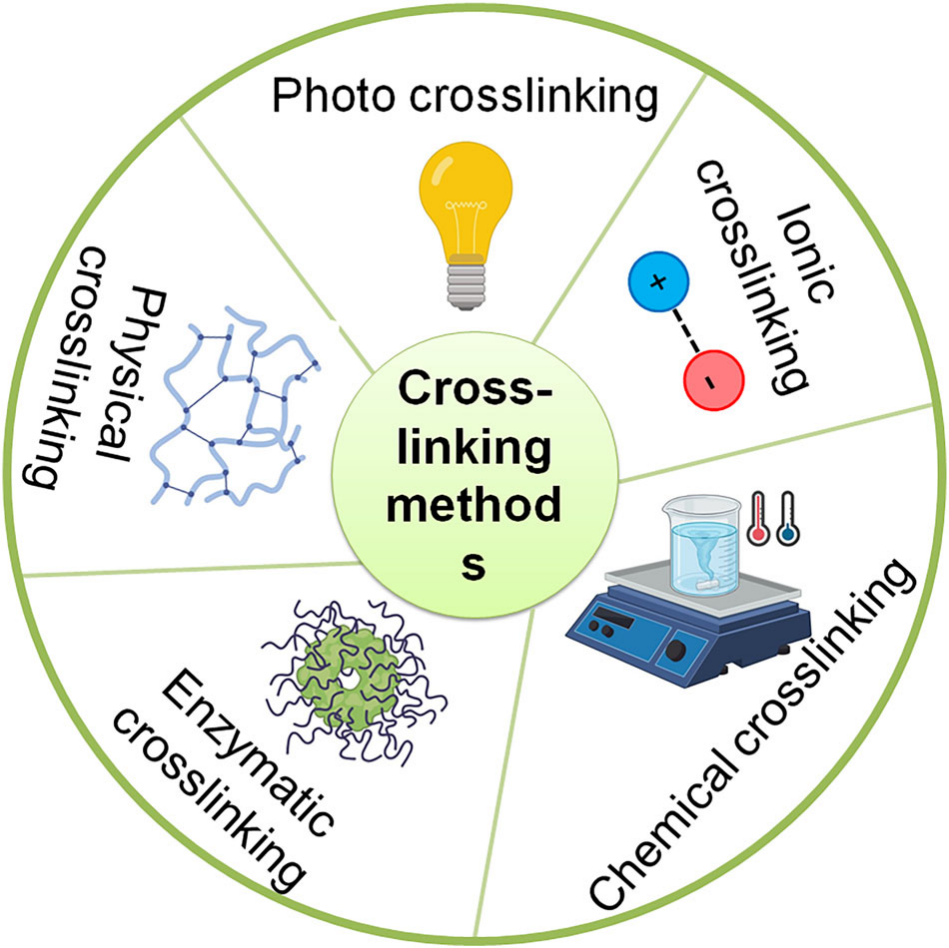
Crosslinking strategies for bioinks in neurobiological research. Depending on the bioink used, crosslinking techniques range from enzymatic, chemical, and ionic to physical and light-aided

**Fig. 10:**
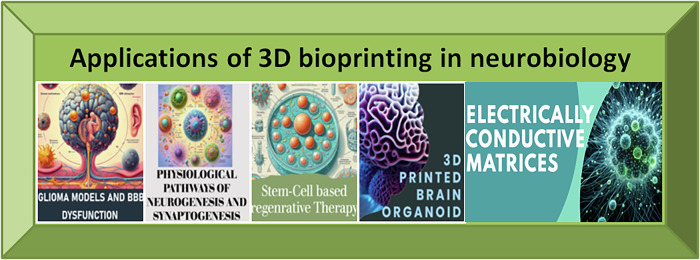
Applications of 3DBP in neurobiology research. The multifaceted utilities of 3DBP encompass creation of cellular models of glioma, organoids and neurovasculature, study of neurogenic and synaptogenic processes, assessment of stem cell-based regenerative strategies, and manufacture of electrically conductive matrices for understanding neuronal electrophysiology

Of note, post-3DBP, some hydrogel-based bioinks need to be induced to form crosslinked networks using chemical, photographic or electrical means, while others are self-organizing based upon temperature changes. Nevertheless, crosslinking is achieved by the covalent bond formation and/or by non-covalent interactions such as hydrogen bridges, hydrophobic and electrostatic interactions [118]. There are several crosslinking strategies for 3DBP (reviewed by [119]) (Fig. 9). The crosslinking step and gelation times are crucial as they substantially impact the physicochemical and biological properties of the bioprinted constructs, which can have tremendous influences on the morpho-functional attributes of loaded living cells. Lastly, appropriate 3D patterning of bioprinted cells is critical for mimicking (patho)physiological settings and the region-specific heterogeneous topographical organization reminiscent of neuronal tissues in the brain and spinal cord [11]. Below, we discuss the advancements in 3DBP in neural tissue engineering, glioma research and electrically conducive platforms to study the responses of neural-like cells to electrical stimulation (Fig. 10).

### Neural tissue engineering

One of the major aims of neural tissue engineering is development of implantable scaffolds for promoting neuronal differentiation, growth and function for regenerative purposes [11]. Biomimetic composites for this purpose should also be biocompatible, have physical characteristics resembling the extracellular matrix of the brain, and should not invoke inflammatory responses [120]. Amongst the first instances of bioprinting of neurons is the study by Xu and coworkers, who directly thermal inkjet-printed embryonic cortical and hippocampal primary neurons in a single layer and confirmed their basic morphology and electrophysiological activities. Further, they successfully created 3D structures of layered neural cells and fibrin gels [121]. Similar 3D neuron-gel composites were fabricated by printing primary embryonic rat neurons and astrocytes onto sodium bicarbonate-assisted collagen gels [122]. In another study, artificial neural tissue comprising of inkjet-printed neural stem cells (C17.2 cells) embedded in collagen hydrogel has been used to study cellular migration towards vascular endothelial growth factor (VEGF)-containing fibrin gels [123]. Collagen has conventionally served as the preferred bioink component for neural tissue engineering (refer to the review by Huang and co-workers; [124]). However, polysaccharides such as agarose, sodium alginate, and carboxymethyl-chitosan have also been tested as bioinks for the creation of porous biocompatible 3D scaffolds for aiding differentiation and growth of morpho-functionally intact neurons [125–127]. Extrusion-based 3DBP of primary rat cortical neurons in sodium alginate and fibrinogen suspension, with transglutaminase and thrombin and CaCl_2_ serving as enzymatic and chemical crosslinkers have been shown to result in fabrication of biomimetic brain tissue-like layered composites in which neurons show appreciable retention of their morpho-functional characteristics [128].

Water-based biodegradable polyurethane gels have also been employed as substrates for bioprinting of neural stem cells, and these cells have been shown to elicit appreciable proliferation and differentiation. In addition, the implantation of these cell-gel composites in zebrafish resulted in significant protection against traumatic brain injury [129]. In a subsequent study, the group showed that extrusion-based 3D co-bioprinting of human fibroblasts and plasmids containing the gene expressing transcription factor Forkhead box D3 (FoxD3) in polyurethane gels successfully resulted in the creation of neural tissue-like 3D composite [130], indicating the utility of 3DBP in neural induction and differentiation, and possibly regenerative therapy.

In several studies, 3DBP of gelatin methacrylate enriched with arginine-glycine-aspartic acid (RGD) peptides has been shown to be effective for creation of an efficient biocompatible scaffold for neural induction and differentiation. For instance, Zhu et al. proposed that a bioink comprising of gelatin methacrylamide, neural stem cells, and bioactive graphene nanoplatelets can be used to fabricate a porous 3D composite using stereolithography in order to induce neural differentiation for nervous tissue regeneration [131]. Similarly, stereolithography-based 3DP of dopamine-containing gelatin methacrylate has been shown to be an excellent scaffold for promoting differentiation of neural stem cells [132]. More recently, Cruz et al. have supported the use of extracellular matrix-based preparation of Geltrex^TM^ in promoting neurogenesis and neurite elongation in neural stem cells in 3D bioprinted gelatin methacrylate-based scaffolds [133]. Gellan gum functionalized with RGD peptides has also been employed as a bioink for encapsulating primary cortical neurons and forming layered brain-resembling 3D micro-constructs. The embedded primary neurons were found to be fully viable and maintained their ability to form complex networks [134]. De-cellularized extracellular matrix preparations, such as Matrigel have also been employed as a scaffold for 3DBP of neural cells. Thus, Li and co-workers fabricated Matrigel-alginate hydrogel scaffolds using extrusion-based 3DBP and found them appropriate for controlled release of brain-derived neurotrophic factor, aiding neural differentiation and growth of ectomesenchymal stem cells [135].

Lastly, 3DBP-based bioengineering has also been proposed as a therapeutic regenerative strategy for spinal cord injuries across multiple studies [136–140]. This is significant as management of peripheral nerve injuries and defects have relied mainly on nerve auto-grafting; however, there are several drawbacks associated with this kind of therapy, including limited supply and donor-site morbidity. In recent years, neural guide conduits have been proposed as alternative therapeutic tools for peripheral nerve injuries [141]. Several novel studies have been focused on applying 3DBP techniques for this aspect of regenerative therapy (reviewed by [142]). For example, stereolithography-based 3D-bioprinted poly(ethylene glycol) diacrylate scaffolds containing polylactic-co-glycolic acid nanoconstructs with the ability for controlled release of neurotrophin, nerve growth factor have shown promising results in promoting neuronal differentiation and neurite outgrowth of PC12 cells [143]. Polycaprolactone scaffolds functionalized by reduced graphene oxide for electrical conductivity synthesized by electrohydrodynamic jet 3DBP were similarly found to induce neuronal differentiation of PC12 cells in vitro [144]. The same group again used electrohydrodynamic jet-based 3D printing for fabrication of polycaprolactone composites and proposed them as effective scaffolds for artificial nerve guide conduits for regenerative therapies against peripheral nerve injuries [145]. Biodegradable polymers such as chitosan [146] and collagen [147] have also been used for the 3D printing of neural guide conduits in recent years. They may have the added benefit of being safer in the long run for regenerative purposes. In an interesting study published recently, Dong and co-workers have used melt electroprinting to fabricate anisotropic microfiber-grid nerve conduits, which showed appreciable regenerative potential upon implantation in vivo in beagle dogs with sciatic nerve defects [148]. Table 5 provides a summarization of the advances in 3DBP for neural tissue engineering.

**Table 5:** Potential applications of 3DBP in neural tissue engineering

S. No.	Construct	3D printing technique	Cells	Major findings	Reference
1.	Collagen-based single layered and fibrin-based 3D micro-fabrications	Inkjet printing	Primary rat hippocampal and cortical neurons (single layered), and NT2 neuronal stem cells (3D constructs)	Bioprinted cells had high viability and retained electrophysiological characteristics	[121]
2.	3D multi-layered collagen hydrogel-based artificial neural tissue	Extrusion printing	Primary rat embryonic neurons and astrocytes	Cells showed normal morphology and high neurite outgrowth and connectivity	[122]
3.	VEGF-releasing fibrin gel constructs with collagen-embedded neuron-like cells	Extrusion printing	C17.2 neural stem cells	High cellular viability, excellent responses to VEGF stimulation for proliferation and migration	[123]
4.	Artificial neural tissue comprised of alginate-carboxymethyl chitosan carrying neuronal and glial cells	Extrusion printing	hiPSCs	Efficient conversion of hiPSCs into cells of specific neuronal subtypes and glial cells	[125]
5.	Artificial neural tissue construct composed of neuronal cell-laden carboxymethyl chitosan	Extrusion printing	Frontal cortical human neural stem cells	Differentiation into functional GABAergic neurons	[126]
6.	Alginate-gelatin-fibrinogen bioink-based artificial brain-resembling tissue construct made of 3 cellular layers and vascular support	Inkjet printing	Rat primary neurons,	High survival rate of electrophysiologically active bioprinted neurons	[128]
7.	3D neuronal bioprints made of PU hydrogel-based thermo-responsive bioink	Inkjet printing	Mouse neural stem cells	Survival and neuromotor rescue of zebrafish with traumatic brain injuries	[129]
8.	Artificial 3D tissue composed of thermo-responsive PU gels expressing FoxD3 transcription factor, and encapsulating human fibroblasts	Extrusion printing	Human dermal fibroblasts	High rates of viability of bioprinted fibroblasts, and their differentiation into neural crest stem-like cells	[130]
9.	3D bioprinted artificial neural tissue made of gelatin methacrylamide and graphene-based nanoplatelets	Stereolithography	Mouse neural stem cells	High survival and density of neural cells with appropriate morphology	[131]
10.	3D bioprinted neural tissue constructs made of gelatin methacrylate-dopamine bioink	Stereolithography	Neural stem cells	Enhanced neuronal differentiation and formation of interconnected network	[132]
11.	3D neural constructs of gelatin methacrylate- Geltrex^TM^ bioink		Mouse cortical astrocytes, hiPSCs, and human neural progenitor cells	Enhanced cellular viability and differentiation of progenitor cells into neurons, and de-differentiation of astrocytes into neural stem cells	[133]
12.	3D in vitro brain-like constructs made up of primary neurons encapsulated in RGD peptide-functionalized gellan gum	Hand-held extrusion-based printing	Mouse primary cortical neurons	Formation of highly interconnected layered neuronal network resembling the 6-layered cortical structure	[134]
13.	3D construct composed of alginate-Geltrex^TM^ bioink encapsulating neuron-like cells, and capable of sustained release of BDNF	Extrusion printing	Rat nasal mucosa-derived ectomesenchymal stem cells	High survival and neuronal differentiation rate of bioprinted stem cells	[135]
14.	Artificial spinal cord-like fabrication composed of gelatin-fibrin- Geltrex^TM^ scaffolds	Extrusion printing	hiPSCs-derived spinal neuronal progenitor cells, and miPSCs-derived OPCs	Formation of functional interacting neuronal networks with directed axonal outgrowths	[136]
15.	3D human and rat spinal cord scaffold made of PEG-gelatin methacrylate or agarose	Micro-scale projection-based printing	Embryonic GFP-expressing neural progenitor cells	Promotion of axonal regeneration in in vivo rats with spinal cord injury	[137]
16.	Spinal cord gelatin methacrylate hydrogel-based stent promoting growth of neuronal and Schwann cells	Inkjet printing	Bone mesenchymal stem cells and rat Schwann cells	Enhanced survival and cellular differentiation, directed axonal growth, and increased intercellular connectivity, resulting in significant neuromotor improvements in rodents with spinal cord injury	[139]
17.	Artificial spinal neural tissue bioprints made of fibrin bioink	Extrusion printing	hiPSC-derived neural progenitor cells	Time-efficient bioprinting resulting in high survival and neuronal differentiation	[140]
18.	3D biomimetic, porous, NGF-releasing nerve guide conduits	Stereolithography	PC12 cells	Increased directed neurite outgrowth of primary cortical neurons	[143]
19.	Synthetic electrically conductive fibrous nerve guide conduits composed of PCL-reduced graphene oxide	Electro-hydrodynamic jet printing	PC12 cells	High viability and neuronal differentiation of the progenitor cells	[144]
20.	3D bioprinted PCL porous nerve guide conduits	Electro-hydrodynamic jet printing	PC12 cells	Enhanced proliferation and neuronal differentiation of bioprinted cells, resulting significant nerve regeneration	[145]
21.	3D printed deacetylated chitosan-based micron track conduit capable of releasing NT-3	DLP	Rat primary Schwann cells	Enhanced and directed growth of Schwann cells, allowing significant repair of peripheral nerves in a rat model	[146]
22.	3D neural tissue electrically conductive constructs composed of collagen-PPy-PCL hydrogels	In-house Inkjet printing	PC12 cells	No cytotoxicity to bioprinted cells	[147]
23.	Artificial biomimetic PLCL-based nerve guide conduits	Melt electroprinting	RSC96 Schwann cells, PC12 cells, and NIH-3T3 fibroblasts	Enhanced nerve regeneration and neuromotor recovery in canine models with sciatic nerve defects	[148]

*BDNF* brain-derived neurotrophic factor, *DLP* digital light processing, *FoxD3* forkhead box D3, *hiPSCs* human induced pluripotent stem cells, *miPSCs* mouse induced pluripotent stem cells, *NGF* nerve growth factor, *NT-3* neurotrophin 3’, *OPCs* oligodendrocyte progenitor cells, *PCL* poly(ε-caprolactone), *PEG* polyethylene glycol, *PLCL* poly(L-lactide-co-ε-caprolactone), *PPy* polypyrrole, *PU* polyurethane, *VEGF* vascular endothelial growth factor

### 3D bioprinted glioma models

Brain cancers, including gliomas, are amongst the most resilient and deadly cancers primarily because their pathogeneses are not completely understood and because of the lack of effective therapies [149]. 3DBP may serve as an excellent practical technique to create biomimetic glioma models resembling the tumor microenvironment, for aiding our understanding of the mechanisms of gliomagenesis and chemotherapeutic resistance (reviewed by [150]; Table 6). Further, they can also be used as platforms for therapeutic evaluation of anti-tumorigenic agents. Scaffolds comprising of sodium alginate, gelatin and fibrinogen with encapsulated glioma cells have been fabricated by extrusion-based 3DBP to study their stemness, angiogenic and endotheliogenic potential, and sensitivity to anti-tumor agents [151–155]. Gelatin methacrylamide [156] and hyaluronic acid [157] have also served as 3D matrices for glioma cells. Cell-cell interactions between the glioma cells and other cell types, such as astrocytes, microglia and macrophages [19, 158].

**Table 6:** 3D bioprinted glioma models

S. No.	3D construct	Cells	Utilities	Reference
1.	Gelatin-alginate-fibrinogen hydrogel scaffold incorporated with glioma stem cells	U118 glioma cells	Identification of molecular players mediating EMT, evaluation of chemotherapeutic potential of temozolomide	[151]
2.	Alginate-collagen hydrogel microfibers incorporating glioma and endothelial cells	U118 glioma cells, HUVEC cells	Assessment of angiogenic pathways in gliomas	[152]
3.	Gelatin-alginate-fibrinogen hydrogel scaffold carrying glioma cells	SU3 and U87 glioma cells	Evaluation of glioma stemness and susceptibility to chemotherapeutics	[153]
4.	3D glioma model comprised of gelatin-alginate-fibrinogen hydrogels as substrate	GSC23 glioma cells	Vasculogenesis and angiogenesis assessment	[154]
5.	Gelatin-alginate hydrogels carrying glioma cells	GSC23 and U87 glioma cells	Understanding transdifferentiation and vascularization of glioma cells, and their therapeutic susceptibility	[155]
6.	3D spheroid glioblastoma model comprised of gelatin-MA hydrogels	U373 glioma cells	Assessment of chemotherapeutic potential of digitoxigenin	[156]
7.	Glioma model comprised of glioblastoma cells and macrophages incorporated into gelatin-MA bioink	RAW264.7 macrophages and GL261 glioblastoma cells	Evaluation of macrophage induction by glioma cells, and therapeutic strategies to inhibit it	[158]

*EMT* epithelial-to-mesenchymal transition, *HUVEC* human umbilical vein endothelial cells, *MA* methacryloyl

Further, BBB dysfunction in gliomas can also be studied in biomimetic 3D printed models (reviewed by [159]). In consistency, 3D microfluidic system-based mimics of BBB have been constructed as suitable models of perusable neurovascular organization. Bioink for this was based upon fibrinogen incorporated with endothelial cells, pericytes, and astrocytes derived from human induced pluripotent stem cells. Interestingly, the model was reported to be self-organizing, with permeability comparable to rodent BBB [160]. Neufeld and co-workers have fabricated nozzle inkjet-based 3D-bioprinted models reproducing the pathophysiological characteristics of perfusable hierarchical vascularization. The model was devised using fibrinogen and gelatin matrices, with lysine and glutamine amino acids serving as ECM-resembling platforms for the formation of intramolecular and intermolecular covalent connections for aiding angiogenesis in the ex vivo tumor microenvironment. Bioink for this model included patient-derived glioblastoma cells, astrocytes and microglia, allowing assessment of the proliferation of these cells by monitoring the induction of the expressions of glial fibrillary acidic protein (GFAP) and ionized calcium binding adapter molecule 1 (IBA-1), respectively [80].

### 3D bioprinted organoids

In vitro 3D brain models, such as organoids have emerged as excellent tools to study the pathophysiological processes underlying brain functions and their dysfunction in diseases [161]. Additionally, these models have the advantage of evaluating human-like patient-specific phenomena, which may not be possible in animal and adherent cultures [162]. Human pluripotent stem cells and induced pluripotent stem cells can be efficiently induced to form neural cells organized as 3D spherical structures. Complementation of brain organoid technology with 3DBP techniques may exhibit significant advantages, particularly for the design of suitable scaffolds to aid cellular differentiation and 3D organization. Use of appropriate bioinks is a prerequisite for fabricating patterned organoids on 3D bioprinted scaffolds [117]. Further, microfluidic integration with 3DBP may allow fabrication of compartmentalized microenvironments for promoting vascularization of organoids. Soft lithography printing techniques are particularly suitable for fabricating microfluidic channel devices for organized network formation of in vitro neurons [163]. For instance, extrusion-based microfluidic printers have been used to construct brain-tissue like 3D cylindrical fabrications composed of neural progenitor cells integrated into fibrin bioink containing polycaprolactone microspheres functionalized with morphogens (such as retinoic acid and purmorphamine). These biomimetic composites were illustrated to stimulate functional activation and maturation of neuron-like cells [164]. Extrusion-based 3DBP has also been integrated with co-axial wet-spinning microfluidic devices to print iPSC-derived neurons and astrocytes in matrigel-alginate hydrogels [165]. Interestingly, 3D printed microfluidic devices have been shown to stimulate interaction of neural cells with vascular elements such as pericytes and endothelial cells to form intricately vascularized brain organoids [166], with potential benefits for the study cerebrovascular pathophysiology. Moreover, patient-derived tumor organoids suitable for pharmacological studies have been fabricated by immersion bioprinting technology using glioma specimen samples [167]. In conclusion, it is anticipated that convergence of organoid culture techniques with 3DBP will serve as a significant and robust tool for creating in vitro brain tissue-like model for evaluation of mechanistic and therapeutic aspects for multiple neuronal disorders, including neurodegeneration, viral infections, epilepsy and neuropsychiatric conditions in a patient-specific manner [168–170]. Table 7 summarizes the utilities of 3DBP in the creation of organoids for brain research.

**Table 7:** 3D bioprinted brain models/organoids

S. No.	3D construct	Cells	Results	Reference
1.	Fibrin-PCL microspheres releasing retinoic acid and purmorphamine	hiPSCs	Promotion of differentiation into neurons capable of responding to acetylcholine and glutamate	[164]
2.	MES-alginate-EDC-NHS microfiber mesh	hiPSCs	Differentiation of cells into neurons capable of synchronized action potential generation	[165]
3.	Cerebral organoids produced on microfluidic chip made of Dental SG	Human pluripotent stem cells-derived neurons, pericytes and endothelial cells	High vascularization of organoids	[166]
4.	Patient-derived glioma organoids constructed using thiolated hyaluronan-methacrylated collagen bioink	Patient-derived glioblastoma cells	Model for high-throughput drug screening assays	[167]
5.	In vitro fibrin gel-based BBB model in 3D printed PDMS microfluidic device	hiPSC-derived endothelial cells, and human brain pericyctes and astrocytes	Self-assembly of vascular networks forming microvasculature with similar permeability as BBB, for the study of drug targeting	[160]

*BBB* blood brain barrier, *EDC* N-(3-dimethylaminopropyl)-N′-ethylcarbodiimide, *hiPSCs* human induced pluripotent stem cells, *MES* morpholineethanesulfonic acid, *NHS* N-hydroxysuccinimide, *PCL* polycaprolactone, *PDMS* polydimethylsiloxane

### Electrically conductive matrices for tissue engineering

A more recent innovation of 3DBP with regards to neurobiology research relates to electrically conductive hydrogels (reviewed by [171]; Table 8). These novel biomaterials have tremendous application in serving as scaffolds for neural tissue-like structures since they can allow electrical stimulation of printed neurons [172]. Indeed, an electrically conductive biopolymer comprising poly(3,4-ethylenedioxythiophene) or PEDOT and polystyrenesulfonate (PPS) has been employed to encapsulate neural stem cells. The neural stem cells were found to be efficiently differentiated into a complex mature network of interacting neurons, indicating their utilities for studying the biological effects of electrical stimulation in engineered biomimetic tissues [173]. Such PEDOT-PSS-PEGDA (polyethylene glycol diacrylate)-based conductive hydrogels created by stereolithography-based 3DP have also been used as a substrate for bioprinting of dorsal root ganglion cells integrated into gelatin methacryloyl matrix. These 3D printed electrically conductive bio-composites were found to significantly enhance neuronal growth and differentiation upon electrical stimulation [174]. Xu and coworkers used 3D printed PEDOT and carboxymethyl chitosan conductive composites as biocompatible and non-toxic platforms with excellent mechanoelectrical properties for the growth of rat PC12 cells, which have neuron-like characteristics [175]. In consistence, other studies have also proposed the applicability of PEDOT-chitosan-gelatin hydrogels as suitable bioinks for the fabrication of brain tissue-like 3D constructs using neural stem cells [176, 177]. Lithography-based 3D printed PEDOT-containing photo-crosslinkable electrically conductive hydrogels have also been shown to have excellent physicochemical properties for supporting the adhesion, growth and differentiation of PC12 cells, allowing the creation of neural tissue-like composites [178]. Other polymers that have been used to fabricate electro-conductive matrices for enhanced adhesion, growth and differentiation of neuron-like cells comprise of alginate-polypyrrole [179], gelatin-polypyrrole [180], alginate-gelatin-polypyrrole-polystyrenesulfonate [181], polyaniline-polyacrylamidomethylpropane sulfonic acid [182], and polycaprolactone-polypyrrole [183], among other polymers. Interestingly, carbon nanofibers have been proposed as agents for generating the ability to conduct electricity in alginate-gelatin hydrogels, and may additionally enhance 3D printability and physical characteristics of elasticity and mechanical durability of the hybrid bioink [184]. Thus, bioprinting of human neural stem cells has also been successfully carried out using biomimetic electro-conductive hydrogels [185], opening up new avenues for neural regeneration-based therapies against brain pathologies. It is clear that because of their abilities to conduct electricity and thereby stimulate neuronal growth, differentiation and migration, electro-conductive hydrogel and aerogels have added advantages for the engineering of 3D printed fabrications resembling brain tissue-like biocomposites which have potential implications for stem-cell based neural regeneration [186], as well as for research focused on understanding the pathways of neuronal differentiation [187].

**Table 8:** 3D printed electrically conducive matrices

S. No.	Conductive polymer	3DP technique	Cells	Results	Reference
1.	PEDOT-PSS	Photolithography	Human neural stem cells	Enhanced formation of functional high-density neural networks	[173]
2.	PEDOT-PSS	Stereolithography	Dorsal root ganglion neuronal cell line (50B11)	Enhanced neuronal differentiation under electrical stimulation	[174]
3.	PEDOT-PSS	Microlithography	PC12 cells	Enhanced cell adhesion, viability, proliferation and neuronal differentiation	[178]
4.	Alginate–PPy	Extrusion printing	PC12 cells	Enhanced cell adhesion, viability, proliferation and neuronal differentiation	[179]
5.	Alginate-gelatin/PPy-PSS	Extrusion-based printing	ATDC-5 cells	High cyto-compatibility of the printed matrix	[181]
6.	PAn-PAAMPSA	Microlithography	NIH/3T3 and PC12 cells	High adherence of NIH/3T3 fibroblasts, but not PC12 neuron-like cells	[182]
7.	PPy-PCL	Electrohydrodynamic jet printing	Human embryonic stem cell-derived neural crest stem cells	High density growth and differentiation of cells into neurons	[183]
8.	Alginate-gelatin-carbon nanofibers	Extrusion-based printing	NIH/3T3 cells	High cellular viability	[184]

*PAAMPSA* polyacrylamidomethylpropane sulfonic acid, *PAn* polyaniline, *PCL* polycaprolactone, *PEDOT* poly(3,4-ethylenedioxythiophene), *PPy* polypyrrole, *PSS* polystyrene sulfonate

In conclusions, 3DBP, complemented with electro-conductive bioinks and microfluidic technology may represent a promising tool for the fabrication of in vitro brain tissue-like organized and patterned structures, which can be used for mechanistic and therapeutic studies [188].

## Conclusions and future perspectives

The rapid advancements in 3DP technologies have significantly impacted neurological sciences, revolutionizing research, diagnostics, and treatment paradigms. From personalized surgical planning and neuroimaging to tissue engineering and drug delivery systems, 3DP has enabled a new era of precision medicine in neurology. One of the most significant contributions of 3DP in neurological sciences is its role in neurosurgery. Patient-specific anatomical models generated from imaging data provide an invaluable tool for preoperative planning, surgical training, and education. These models help clinicians to visualize complex anatomical structures, simulate surgical interventions, and reduce intraoperative complications. The ability to print customized implants and prosthetics has further improved patient-specific care, particularly in cranioplasty and spinal reconstruction, ensuring better functional and aesthetic outcomes. The integration of 3DP in neuroimaging has also shown tremendous promise. Anthropomorphic phantoms that mimic human tissues in terms of anatomical and radiological properties have been employed to refine imaging techniques such as MRI, CT, and TUI. These phantoms assist in the standardization and optimization of imaging protocols, ensuring accurate diagnosis and treatment planning for neurological conditions. Furthermore, the application of 3DP in creating cerebrovascular models has enabled the study of stroke, aneurysms, and arteriovenous malformations with improved precision, ultimately aiding in the development of targeted therapeutic strategies. The role of 3DP in pharmaceutical and therapeutic applications is another domain that has gained considerable attention. The fabrication of patient-specific drug delivery systems, such as customized nasal casts for nose-to-brain drug delivery, has provided new avenues for targeted treatment of neurological disorders. These 3D printed drug delivery platforms have demonstrated the potential to bypass the BBB, improving the bioavailability of therapeutic agents and reducing systemic side effects.

3DBP techniques have also facilitated several aspects of neurobiological research. Fabrication of biomimetic scaffolds that support neuronal growth and differentiation is one prominent application. These 3D bioprinted constructs have been employed to model neural tissues, study neurodegenerative diseases, and explore potential regenerative therapies for spinal cord injuries and peripheral nerve defects. The development of electrically conductive bioinks has further enhanced the potential of 3DBP in neural tissue engineering by providing a platform for studying neuronal electrophysiology and facilitating neural network formation. Moreover, 3D bioprinted organoids and glioma models have emerged as robust platforms for studying tumor microenvironments, screening anticancer drugs, and developing personalized treatment strategies for brain cancers.

Hence, it is clear that integrating 3DP/BP with neurological sciences has enabled significant advances in multiple aspects of clinical and biomedical research, offering opportunities to understand and manage brain pathophysiological states. Despite these remarkable advancements, several challenges remain in fully integrating 3DP and 3DBP into routine clinical and research practices. Material limitations, printing resolution, scalability, and regulatory considerations are some of the primary concerns that need to be addressed. Additionally, development of novel bioinks that closely mimic the extracellular matrix, ensure cell viability, and support long-term cellular function is crucial for advancing neural tissue engineering applications. Similarly, improvements in bioprinting resolution and multi-material printing capabilities will enhance the fabrication of complex neural architectures with functional integration. Regulatory and ethical considerations also play a vital role in the adoption of 3DP in neurology. Standardization of printing protocols, quality control measures, and long-term safety evaluations are essential to ensure the clinical translation of 3D bioprinted constructs. Further, independent studies are needed to validate findings from 3D models. Other significant challenges are also associated with further expansion of 3DBP in understanding and managing neuropathologies, particularly neuropsychiatric conditions involve complex multifactorial origins that are difficult to fully capture in vitro. Similalrly, therapeutic efficacy demonstrated ex vivo must still be proven in vivo to ensure agents can effectively access and target specific nervous system sites. Collaborative efforts between biomedical engineers, neuroscientists, regulatory agencies, and healthcare professionals are hence required to establish guidelines that facilitate the safe and effective use of 3DP and 3DBP technologies in neurological science research.

Moving forward, the future of 3DP and 3DBP in neurological sciences is poised for further innovation and expansion. The convergence of 3DP with artificial intelligence, machine learning, and bioinformatics holds promise for developing intelligent and adaptive neuroprosthetic devices. Similarly, integration of 3D printed models with virtual and augmented reality technologies can enhance surgical simulation, medical education, and patient engagement. Additionally, the advancement of 4D printing, which incorporates dynamic and responsive materials, may revolutionize the design of smart implants and neural interfaces. Interdisciplinary collaboration will be key to unlocking the full potential of 3DP in neurology. By fostering partnerships between engineers, neuroscientists, clinicians, and regulatory bodies, we can accelerate the development and adoption of 3DP and 3DBP technologies for neurological applications. Continued investment in research and innovation, along with robust regulatory frameworks, will pave the way for translating these groundbreaking technologies into clinical practice, ultimately improving patient care and advancing our understanding of the nervous system.

In conclusion, 3DP and 3DBP have ushered in a transformative era in neurological sciences, offering unprecedented opportunities for personalized medicine, research advancements, and therapeutic interventions. While challenges remain, the progress made thus far underscores the immense potential of these technologies in revolutionizing neurology. By addressing current limitations and embracing future innovations, 3DP and 3DBP will continue to shape the future of neurological research and healthcare, ultimately improving the lives of patients suffering from neurological disorders. One can hope that in coming years, continued multidisciplinary efforts will imperatively advance this field and translate insights into clinical applications that may one day effectively treat/cure neurological disorders.
